# Biological properties of carotenoids extracted from *Halobacterium halobium* isolated from a Tunisian solar saltern

**DOI:** 10.1186/1472-6882-13-255

**Published:** 2013-10-04

**Authors:** Molka Abbes, Houda Baati, Sonda Guermazi, Concetta Messina, Andrea Santulli, Neji Gharsallah, Emna Ammar

**Affiliations:** 1 Ecole Nationale d’Ingénieurs de Sfax, UR: Etude et Gestion des Environnements Côtier et Urbain, Université de Sfax, B.P. 1173, 3038 Sfax, Tunisia; 2 Laboratoire de Génétique Moléculaire des Eucaryotes, Centre de Biotechnologie de Sfax, B.P. 1177, 3038 Sfax, Tunisia; 3 Department BIONEC, Section Biochemical Science, Marine Biochemistry Laboratory, University of Palermo, Via Barlotta 4, 91100 (TP), Italy; 4 Marine Biology Institute, Consorzio Universitario della Provincia di Trapani, Via Barlotta 4, 91100 (TP), Italy; 5 Faculté des Sciences de Sfax, Laboratoire de Biotechnologie Microbienne, B.P. 802, 3018 Sfax, Tunisia

**Keywords:** *Archaea*, *Halobacteria*, Solar saltern, Carotenoids, HepG2 human cancer cells

## Abstract

**Background:**

Bioactive molecules have received increasing attention due to their nutraceutical attributes and anticancer, antioxidant, antiproliferative and apoptosis-inducing properties. This study aimed to investigate the biological properties of carotenoids extracted from *Archaea*.

**Methods:**

Halophilic *Archaea* strains were isolated from the brine of a local crystallizer pond (TS7) of a solar saltern at Sfax, Tunisia. The most carotenoid-producing strain (M8) was investigated on heptoma cell line (HepG2), and its viability was assessed by the MTT-test. The cells were incubated with different sub-lethal extract rates, with carotenoid concentrations ranging from 0.2 to 1.5 μM. Antioxidant activity was evaluated through exposing the cells to sub-lethal extract concentrations for 24 hours and then to oxidative stress induced by 60 μM arachidonic acid and 50 μM H_2_O_2_.

**Results:**

Compared to non-treated cells, bacterial carotenoid extracts inhibited HepG2 cell viability (50%). A time and dose effect was observed, with cell viability undergoing a significant (*P* < 0.05) decrease with extract concentration. After exposure to oxidative stress, control cells underwent a significant (*P* < 0.05) decrease in viability as compared to the non-treated cells.

**Conclusions:**

The bacterial extracts under investigation were noted to exhibit the strongest free radical scavenging activity with high carotenoid concentrations. The carotenoid extract also showed significant antiproliferative activity against HepG2 human cancer cell lines.

## Background

In hypersaline environments, interest in living microbes i.e. halophilic microorganisms, has increased by the recent discoveries of new taxa useful for several biotechnological applications and processes, including biopolymers, biosurfactants, exopolysaccharides, compatible solutes, and bioactive compounds (carotenoids, anti-tumor and antimicrobial substances, etc.) [1,2]. During the last few decades, the analysis of microbial diversity has shifted from cultivation-dependent approaches to 16S rRNA-based cultivation-independent approaches [3], which led to the discovery of new microbial taxa. In fact, various molecular culture independent techniques have been used to characterize the microbial communities in hypersaline environments [4-6].

Some of the bacterial and archaeal communities in the brines of Tunisian solar salterns were previously investigated using culture-independent molecular approaches wherein their properties and activities were compared at different salt concentrations [7]. Several moderately halophilic strains were also isolated and investigated for their phenotypic characteristics, phylogenetic affiliation and enzymatic activities [8]. As far as the *Archaea* population is concerned, most of the 16S rRNA gene sequences so far obtained from Tunisian crystallizer ponds were affiliated with the family of *Halobacteriacaea*[7]. Reports have also shown that halophilic *Archaea* with red carotenoid pigments improved brine light absorption and promoted evaporation by increasing temperature [9].

Carotenoids have received increasing attention for they are the most abundant pigments in nature, with carotenoids from marine origins being structurally different from those found in terrestrial environments. In halophilic *Archaea*, bacterioruberin is considered as the major representative of the C50 carotenoids [10]. Several reddish food products, red *Archaea*, and carotenoids, a group of lipid-soluble compounds responsible for the yellow and red colors in many plants, have been demonstrated to be effective in the prevention of various chronic illnesses, including skin cancer and cardiovascular diseases [11]. Carotenoids are also widely distributed in nature and have a considerable potential for application as nutraceuticals and dietary antioxidants [12].

**Figure 1:**
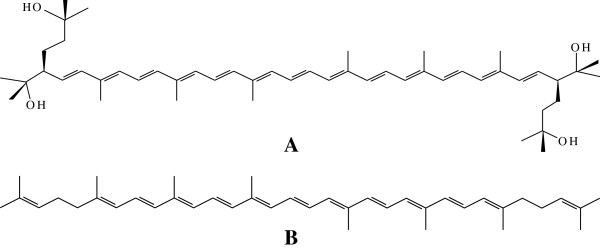
Structure of bacterioruberin (A) and lycopene (B) pigment [13].

Furthermore, bacterioruberin [13] is known to contain 13 pairs of conjugated double carbon bonds, endowing biological tissues with effective hydroxyl free-radical scavenger power and singlet oxygen quenching activity (Figure 1). This pigment can protect *Halobacteria* from fatal injuries under intensive light [14,15] and confers bacteria with resistance to oxidative DNA damage from radiography, UV-irradiation, and H_2_O_2_ exposure [16]. Bacterioruberin also has other equally important roles for membrane fluidity, including its function as a water barrier and responsibility for the permeability of oxygen and other molecules, thus enhancing bacterial survival in hypersaline and low-temperature environments [17-19].

Considering the promising properties and attributes of carotenoids, increasing attention has recently been given to the discovery of novel natural carotenoids with practical functional applications in the prevention of human health diseases [20-22]. In this context, carotenoids and flavonoids have been reported to form complexes with metal ions altering or inhibiting metabolic pathways [23]. Carotenoids from terrestrial origins, such as β-carotene and lycopene, have particularly been investigated as cancer preventive agents [24-27]. Continuing with this line of research, the present study was undertaken to investigate the antioxidant and antiproliferative properties of a carotenoid extract (CE) from brine solar saltern halophilic *Archaea* in non-cellular and in cellular systems, respectively, using human (HepG2) hepatoma cell lines.

## Methods

### Brine sample collection

The brine sample used in this work was aseptically collected from a local crystallizer pond (TS7) of a solar saltern at the city of Sfax, Tunisia (Central-Eastern coast of Tunisia, about 34°39’N and 10°42’E). The sample was the average of ten sub-samples spanning over the whole pond. The representative sample was centrifuged at 12000 g for 10 min, washed with sterile phosphate-buffered saline (PBS) containing (per liter): KCl, 0.2 g; NaCl, 8 g; KH_2_PO_4_, 0.2 g; Na_2_HPO_4_.12H_2_O, 29 g (pH 7.4), and then stored at –20°C until further use. The physical and chemical parameters of the TS7 brine were determined according to the method described by Wang et al [26].

### Isolation procedure

Isolations were performed on a complex medium (DSC-97) containing (per liter): yeast extract, 10 g; casamino acids, 7.5 g; NaCl, 250 g; MgSO_4_.7H_2_O, 20 g; KCl, 2 g; and trisodium citrate, 3 g [28]. The pH was adjusted to 7.4 [29]. Dilutions were spread on DSC-97 agar plates. After incubation at 37°C in a salt saturated atmosphere for 15–20 days, grown red colonies were selected and purified. Different colonies were purified by at least four streaking rounds on fresh agar plates until a pure colony was obtained. The isolates were preserved in 40% glycerol (w/v) at -80°C for further use. Colonies were streaked on a solidified medium containing chloramphenicol at 20 mg/l and used for the determination of the *Archaea* halophilic genus of the isolates. The cultures were incubated for ten days at 37°C, and the developed strains were submitted for further molecular identification assays [30].

### Hyper carotenoid-producing strains

The isolates were incubated in 500-ml erlenmeyer flasks containing 100 ml of DSC-97 broth at 37°C and 240 rpm for 7 days. Optical density at 660 nm was measured with a spectrophotometer (Hitachi U 2001, Tokyo).

### Bacterial DNA preparation and 16S rRNA amplification

A Quiagen DNA kit was used for genomic DNA extraction. The extracted DNA was then visualized by electrophoresis on 1% agarose gel with ethidium bromide staining. The 16S rRNA gene was amplified by PCR using TaKaRa Ex Taq^TM^ (2.5 units, Promega) in 50 μl reaction buffer, containing 2 mM of each dNTP (dATP, dTTP, dGTP, dCTP), 20 μM of each primer, and 5 μl of 10x Ex *Taq* buffer^TM^. The primers used were archaeal-specific primer 21 F [31] combined with the universal reverse primer 1390R [32]. The PCR thermal profile was as follows: initial denaturation at 94°C for 2 min and 30 cycles consisting of denaturation at 94°C for 30 sec, primer annealing at 59°C for 30 sec, and extension at 72°C for 1.5 min. The final elongation step was extended to 10 min. Under these conditions, a single PCR product of 1.4 kb was analyzed on 1% agarose gel stained with ethidium bromide and visualized under ultra-violet trans-illumination.

### Phylogenetic analysis of 16S rRNA

The resulting 1.4 kb 16S rRNA gene sequence obtained was compared to those available at the GenBank and EMBL databases using advanced BLAST searches at the National Center for Biotechnology Information (NCBI). Sequences were analyzed using the ARB software package (http://www.arb-home.de, 2005 version) for phylogenetic analysis.

### Nucleotide sequence accession numbers

The sequence data obtained in this study has been submitted to EMBL/GenBank databases under Accession Number HF546975.

### Carotenoid extraction

At the stationary growth phase, 50 ml of the culture were centrifuged (20 min, 0°C, 6000 rpm). The cell pellet was separated from the supernatant and extracted in dark with acetone (100 ml) containing the antioxidant butylhydroxytoluene (BHT) (50 mg). The solution was then centrifuged for 10 min at 4°C and 6000 rpm. After that, the acetone cell suspension was mixed with hexane (5 ml) and NaCl 25% aqueous solution (5 ml) until separation was achieved. The aqueous acetone layer was extracted in a second step with hexane. After agitation for a few minutes, the hexane extract containing carotenoids was dried in a rotary vacuum evaporator, weighed, and resuspended in ethanol [33]. The CE was scanned in the wavelength region of 400-600 nm using a spectrophotometer. The total carotenoid concentration was calculated using the value obtained at the maximum absorption wavelength (K max = 489 ± 2 nm). The absorption coefficient value used was 2660 as recommended by Britton for halophilic bacteria [34].

### Human heptoma cell culture

The human hepatoma (HepG2) cell line used in this work was purchased from Interlab Cell Line Collection (Genova, Italy). The cells were grown as a monolayer in an RPMI 1640 medium supplemented with 10% (v/v) fetal bovine serum (FBS), 1.0 mM sodium pyruvate and 2 mM l-glutamine at 37°C in a humidified atmosphere of 95% air and 5% CO_2_[35]. After 24 h of growth, the cells were transferred into a 96-well plate (10^4^ cells/well) and treated with the CE for 24 and 48 h, respectively. The CE was dissolved in ethanol and served as a stock solution that was later diluted to have a final solvent concentration of less than 0.1% (v/v) in the medium. The effects associated with ethanol withdrawal on the HepG2 cells were previously assessed. The CE was tested at 0.05 – 0.1% and 0.2% (weight of total carotenoids extract/volume).

### Cytotoxicity measurement

The cell morphology was defined through optical microscopic observations (Olympus x50). The MTT-assay was used to evaluate the CE effect on HepG2 cell viability, and the results were expressed as viable cell percentages with respect to the control. Approximately 10^4^ cells/well were seeded onto a 96-well plate and allowed to adhere for 24 h. Three replicates of each plate were incubated with different sub-lethal concentrations of CE (0.2, 0.5 and 1.5 μM), and viability was recorded at 24 and 48 h [36,37]. After treatment, the medium was removed, and 20 μl of 11 mg/ml solution of MTT in PBS were added to each well. The plate was then incubated for 2 h at 37°C. Finally, the medium was removed, and 200 μl of the lyses solution were added in each well to solubilize the blue formazan. Dye absorbance was measured at 570 nm.

### Oxidative stress induction

The CE antioxidant effect was evaluated using cells treated with extract sub-lethal concentrations, and oxidative stress was induced by arachidonic acid and hydrogen-peroxide (H_2_O_2_). After 24 hours of reaction, the medium was replaced by 200 μl of serum-free MEM containing arachidonic acid (60 μM) [36] or H_2_O_2_ (50 μM) as a pro-oxidant [38], and incubation was extended to 24 h. The CE protective effect against cell viability reduction as induced by oxidative stress was also assessed using the MTT assay as previously described.

### Statistical analysis

Statistical analysis was performed using the Statistical Package for Social Sciences (SPSS 11 for Windows). Statistical significance was defined for *P <* 0.05.

## Results and Discussion

### Hyper carotenoid –producing Halobacteria isolation and screening

The red-pink and viscous brine used for the isolation of carotenoid-producing Halobacteria was collected from a local crystallizer pond (TS7) at the solar saltern of Sfax, Tunisia. The results from the physico-chemical analyses of the samples are reported in Table 1. The pond from which the samples were collected had a rather basic pH and a high total salt concentration, which enhance sodium chloride precipitation. Density was, therefore, important, fitting the model previously established by Baati et al [27].

**Table 1:** Physico–chemical characteristics of brine TS7 pond (July 2009)

**Pond**	**TS7**
***Physical parameters***	
Temperature (°C)	34.0 ± 6.0
pH	8.30 ± 0.7
Densité	1.224 ± 0.006
Turbidity (NTU)	45.1 ± 0.5
Reduction potential (mV)	-38.6 ± 0.4
Salinity (%)	28.43 ± 0.2
***Major cations and anions (g/l)***	
Na^+^	95.00 ± 0.95
Mg^2+^	19.954 ± 0.098
K^+^	5.780 ± 0.051
Ca^2+^	0.260 ± 0.008
Cl^-^	188.54 ± 3.10
SO_4_^2-^	28.65 ± 0.21
HCO_3_^-^	0.451 ± 0.082
C0_3_^--^	0.000

The strains grown on DSC-97 agar plates were noted to produce pink-red shaded colonies (red, blood-red, brick-red, orange-red, pink, bright-pink and pale-pink). In all, ten red colonies of Halobacteria were purified. The growth of each isolate was tested in a medium containing chloramphenicol to distinguish between *Archaea* and *Bacteria* domains. The strains that were able to grow on this medium were taken to belong to the *Archaea* domain [30].

The carotenoid production yields of the isolates ranged between 5.66 and 7.63 mg/l. The findings revealed that the highest concentrations of carotenoids were achieved with strain M8. This strain was a cocci-shaped, non motile, Gram-negative bacterium occurring individually, in pairs, or in irregular clusters. After incubation on an agar medium (37°C for 15 to 20 days), the colonies were red-orange, opaque, smooth, and slightly rounded, with a diameter ranging between 0.1 and 0.3 mm. These characteristics are similar to those of the *Halobacterium* genus. Most isolates required at least 2.5 M NaCl, with an optimum ranging between 3.5 and 4.5 M and 0.1 and 0.5 M Mg^2+^[39].

**Figure 2:**
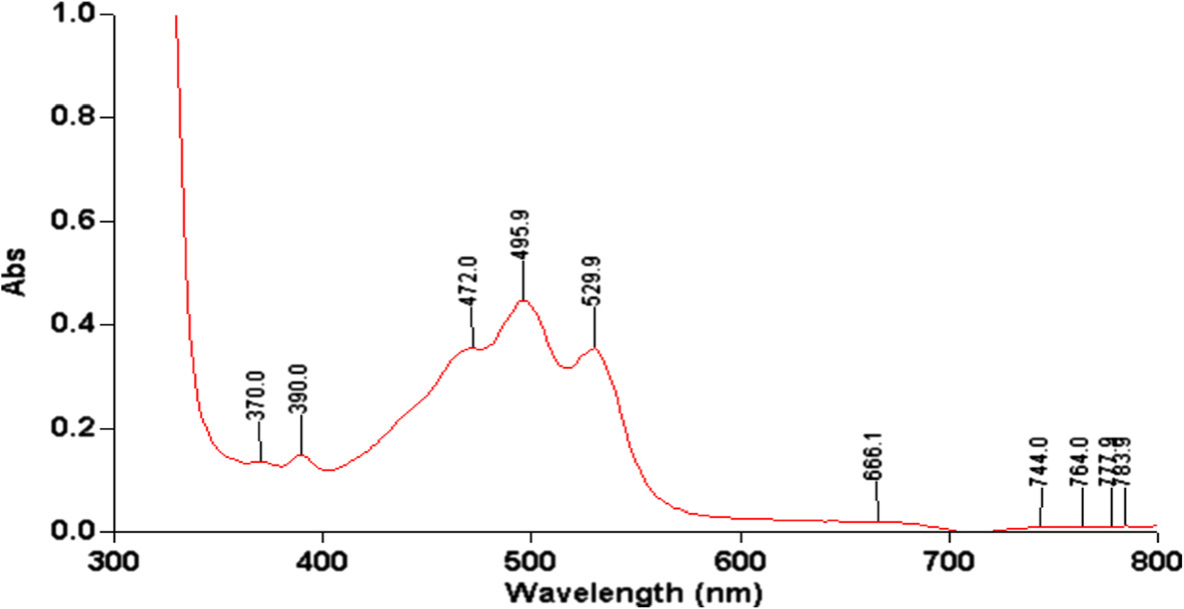
**Absorbance spectrum of *Halobacterium halobium *M8.**

The correlation between the concentrated seawater color (brine) and viable and cultivable bacteria was previously investigated by Donadio et al [40] who reported that when the color intensity increases, the viable and cultivable bacteria concentrations, enumerated on plate count agar, increases. Furthermore, the red color of most members of *Halobacteriaceae* was used as an easily recognizable feature to discriminate between archaeal and bacterial members of the prokaryotic community [41]. As far as absorption is concerned, the CE from the strains was analyzed by scanning spectrophotometric absorption. The results showed that they had a similar absorption spectrum (Figure 2). Kelly et al [42] suggested that bacterioruberin is a characteristic carotenoid from halophilc *Archaea*. Britton [34] reported that the spectral peaks exhibited by bacterioruberin and its derivatives were characteristic of red carotenoids, with nearly identical absorption maxima at 467, 493 and 527 nm. Later, Asher and Ohta [43] found that all the red Halobacteria strains, isolated from the Egyptian seawater evaporation pond, exhibited identical carotenoids absorption spectra, which were characterized by maximal absorption rates at 493 and 527 nm, with a broad shoulder at 467 nm. Although no pure standard is currently available for use in comparative studies, the findings of this study and the data reported in the literature provide strong support that these peaks are indicative of a bacterioruberin-like carotenoid.

### Phylogenetic tree construction

**Figure 3:**
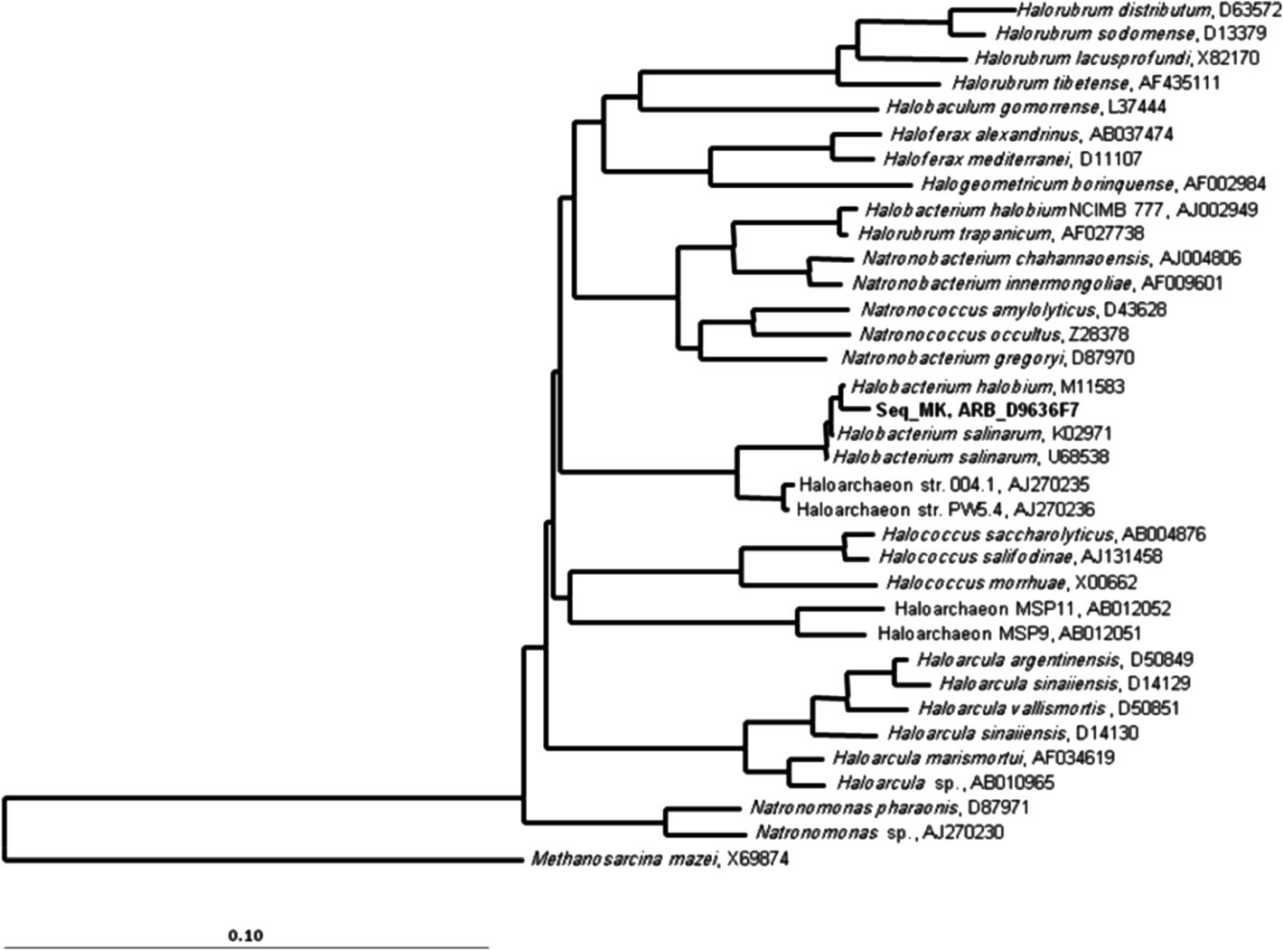
**Phylogenetic tree of the M8 strain.** Phylogenetic relationships between the M8 strain 16S rRNA sequences and other related archaeal sequences previously published in the databases. The phylogenetic tree was built by Neighbour-joining method using the ARB software package. The scale bar corresponds to a 10% estimated difference in nucleotide sequence positions. *Methanosarcina mazei* (X69874) was used as an outgroup.

The phylogenetic position of strain M8 was determined based on 16S rRNA gene sequencing. The best BLAST hits were recorded with sequences from the *Halobacteriales* order and, more precisely, with representatives of the *Halobacterium* genus, suggesting that M8 would be a representative of this group. Among sequences from cultivated organisms, the sequence *Halobacterium halobium* M11583 displayed the highest similarity (99%) with M8, suggesting a close relationship between these strains (Figure 3).

### Carotenoids extract effect on cell viability

**Figure 4:**
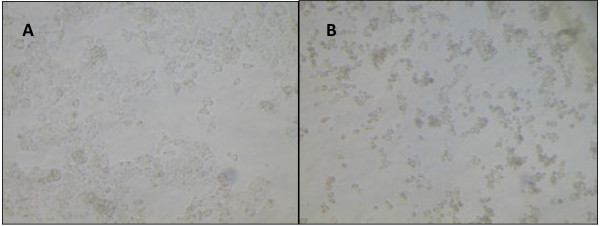
**Morphological changes of Hep-G2 cells treated with the bacterial extract.** The cells (10^4^ cells/well) were incubated in the presence of medium alone **(A)** and CE (0.2-0.5 μM) **(B)** for 48 h. Morphological changes were observed by microscopy (x50).

The HepG2 cells treated with increased concentrations of the M8 CE showed a significant (*P* < 0.05) decrease in cell viability in a time and dose dependent way. In fact, at low CE concentrations (0.2 - 0.5 μM), no significant decreases in cell viability were recorded, as compared to the control, despite the morphological modifications revealed by optic microscopy (Figure 4). The treatment of HepG2 cells with CE was also noted to induce a decrease in cell sizes. This observation is in accordance with the significant change observed for HepG2 cell morphology after 24 h of treatment with 0.5-1 mg/l (0.9 - 1.8 μM) of the *Haloferax Mediterranean* (hmERP) extract [44]. This extract was noted to induce cell death in a dose-dependent way. The hmERP exposure was also associated with the cell morphology change, with a HepG2 shrinkage.

### *In vitro* antioxidant and pro-oxidant activities of carotenoid extract

**Figure 5:**
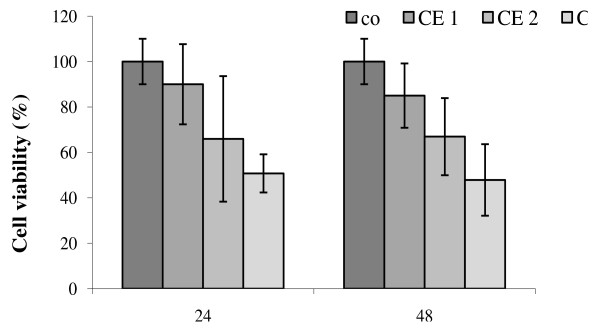
Effect of carotenoid extract in HepG2 cells on cell viability: co: Control; CE: carotenoids extract, 1 : 0.2 μM, 2 : 0.5 μM, 3 : 1.5 μM.

A significant (*P* < 0.05) decrease in cell viability was observed for high CE concentrations (1.5 μM), reaching up to 50% (Figure 5). Most studies dealing with cell lycopene treatment demonstrated a decrease in cell proliferation with the increase in extract concentration. Lycopene treatment has recently been shown to inhibit cell viability of HepG2 by 30%. The lowest cell viability reduction was observed in HepG2 cells as compared to human colon (HT-29) and breast cancer (MCF-7) cell lines [45]. These data indicate that the lycopene effect was cell-specific, time and dose dependent, as well as time-demanding for it would require a relatively long incubation time in most cell lines. In other cases involving HepG2 treatment with 1 and 10 μM lycopene doses, however, cell viability was reported to decrease after 24 h incubation [46]. Other reports showed that lycopene can induce cell cycle inhibition at the first phase. Park et al [47] reported that the growth of human hepatoma cells (Hep3B) inhibition ranged from 20 to 50% by lycopene at physiological concentrations lower than 0.2 μM.

Previous studies demonstrated the anti-proliferation properties of fifteen carotenoids against prostate cancer cells [48] and their potent growth-inhibitory activity in several tumor cells, including colon, melanoma, prostate, oral, lung, and breast cancer cells. They were also reported to enhance the chemotherapeutic cytotoxicity of the cell lines. In fact, the cancer preventive potential of carotenoids has been demonstrated in several studies involving cultured cells and experimental animals. Carotenoids have been shown to suppress the *in vitro* cancer cell propagation by inducing differentiation and apoptosis, thus enhancing gap-junctional communication and inhibiting the cell cycle [49-52].

**Figure 6:**
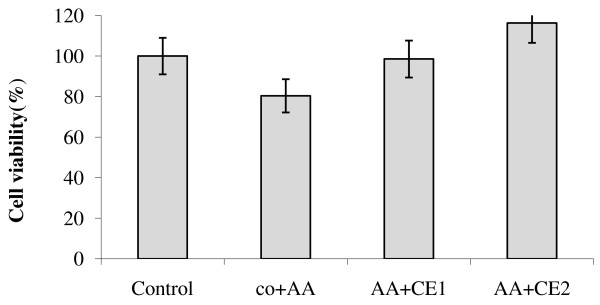
HepG2 cell viability after exposure to carotenoids extract and oxidative stress with arachidonic acid (AA).

**Figure 7:**
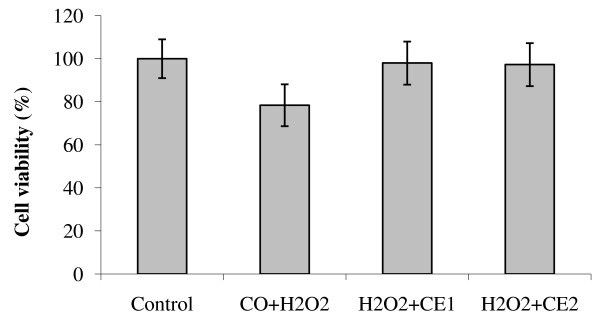
**HepG2 cell viability exposed to carotenoids extract and oxidative stress with hydrogen peroxide (H**_**2**_**O**_**2**_**).**

Among the various defense strategies, carotenoids are most likely involved in the scavenging of two of the reactive oxygen species, singlet molecular oxygen (^1^O_2_) and peroxyl radical [53]. In this study, control cells exposed to oxidative stress with arachidonic acid and H_2_O_2_ separately showed a significant (*P* < 0.05) reduction in viability as compared to non-exposed cells. The cells pre-treated with the lower non lethal concentrations of CE (0.2 - 0.5 μM) for 24 hours were, on the other hand, noted to exert a significant (*P* < 0.05) protective effect against cell death induced by oxidative stress (Figure 6 and Figure 7). The highest rate of increase in the cell viability of HepG2 incubated with arachidonic acid was recorded with 0.5 μM of CE. In fact, Xu et al [35] reported that lycopene attenuates the toxicity of arachidonic acid in HepG2 cells. Under some circumstances, carotenoids were also shown to act as cellular antioxidants. β-carotene was, for instance described to suppress the up-regulation of haem oxygenase-1 gene expression provoked by UVA exposure in human dermal fibroblasts (FEK4) in a dose-dependent manner [54]. Teodoro et al (2012) showed that only HepG2 cell lines responded to lycopene by a reduction in cell numbers after 24 h of incubation at 1 and 10 μM doses. HepG2 cell line proliferation was also reported to decrease at 1, 3, and 5 μM, but after 96 h of incubation [45].

## Conclusion

The present study concluded that the Haloarchaea isolated from the solar saltern of Sfax, Tunisia have a promising biopotential that might open new promising opportunities for the development of potent bioactive agents. The halophilic *Archaea* presented in this work can also be used as potential sources for antitumor and antioxidant metabolites. Further studies, some of which are currently underway in our laboratories, are needed to further characterize the bacterial strain and investigate the pathways triggering apoptosis in cancer cell exposed to CE.

## Abbreviations

ROS: Reactive oxygen species; PBS: Phosphate buffered saline; DSC 97: Complex medium; PCR: Polymerase chain reaction; RPMI 1640: Roswell Park Memorial Institute medium; FBS: Fetal bovine serum; CE: Carotenoids extract; MTT: 3-(4,5-dimethylthiazol-2-yl)-2,5-diphenyltetrazolium bromine; MEM: Minimum essential medium.

## Competing interests

The authors declare that they have no competing interests.

## Authors’ contributions

MA, HB, CM and EA conceived and designed the experiments. MA performed the experiments. MA, HB, SG and CM analyzed the data. EA, NG and AS contributed with reagents and materials as well as analysis tools. MA, HB and EA wrote the paper. All authors read and approved of the final manuscript.

## Pre-publication history

The pre-publication history for this paper can be accessed here:

http://www.biomedcentral.com/1472-6882/13/255/prepub
